# Thermal Imaging and Dimensionality Reduction Techniques for Subclinical Mastitis Detection in Dairy Sheep

**DOI:** 10.3390/ani14121797

**Published:** 2024-06-15

**Authors:** Christos Tselios, Dimitris Alexandropoulos, Christos Pantopoulos, Giorgos Athanasiou

**Affiliations:** 1 Industrial Systems Institute, Athena Research Center, Patras Science Park Building Platani, 26504 Patras, Greece; 2 Department of Materials Science, University of Patras, 26504 Rion, Greece; 3 Powermilk, 81107 Kalloni Lesvos, Greeceinfo@powermilk.gr (G.A.)

**Keywords:** dimensionality reduction, thermal imaging, livestock management, subclinical mastitis

## Abstract

**Simple Summary:**

Subclinical mastitis is a common and economically significant disease that affects dairy sheep production. This study aims to develop and evaluate an advanced algorithmic approach that integrates thermal imaging, statistical texture analysis, and t-distributed stochastic neighbor embedding (t-SNE) to accurately detect subclinical mastitis in dairy sheep. This approach focuses on improving the accuracy and reliability of non-invasive subclinical mastitis detection by using more sophisticated algorithmic procedures than traditional temperature differential methods, thereby enhancing livestock management and animal health monitoring.

**Abstract:**

Subclinical mastitis is a common and economically significant disease that affects dairy sheep production. Thermal imaging presents a promising avenue for non-invasive detection, but existing methodologies often rely on simplistic temperature differentials, potentially leading to inaccurate assessments. This study proposes an advanced algorithmic approach integrating thermal imaging processing with statistical texture analysis and t-distributed stochastic neighbor embedding (t-SNE). Our method achieves a high classification accuracy of 84% using the support vector machines (SVM) algorithm. Furthermore, we introduce another commonly employed evaluation metric, correlating thermal images with commercial California mastitis test (CMT) results after establishing threshold conditions on statistical features, yielding a sensitivity (the true positive rate) of 80% and a specificity (the true negative rate) of 92.5%. The evaluation metrics underscore the efficacy of our approach in detecting subclinical mastitis in dairy sheep, offering a robust tool for improved management practices.

## 1. Introduction

Subclinical mastitis poses a significant challenge in dairy sheep production, impacting milk yield, quality, and overall herd productivity [1,2]. Environmental factors such as poor hygiene in milking parlors and housing facilities contribute to its prevalence in Greece, facilitating the spread of infectious agents [3,4]. Infrared thermography (IRT) has emerged as a promising tool for early mastitis detection, utilizing infrared cameras to capture udder heat emissions and identify temperature changes indicative of inflammation. However, complex data patterns and structures present challenges in analyzing thermal images.

Using thermal imaging has led to significant results in detecting subclinical mastitis in dairy sheep and cows. Kunc et al. [5] proposed IRT as a non-invasive tool for evaluating the milking process, including its potential for early mastitis detection in dairy cows. Colak et al. [6] demonstrated a strong correlation between the udder skin surface temperature (USST) and the results of the California mastitis test (CMT) for 94 cows. Polat et al. [7] proposed cut-off values for udder skin surface temperature as indicators for differentiating between healthy and subclinically mastitic udder quarters based on somatic cell count (SCC) thresholds, demonstrating high sensitivity and specificity for mastitis detection. Sensitivity measures the ability of thermal imaging to correctly identify animals with the disease and specificity measures the ability to correctly identify animals without the disease. Specifically, for quarters with SCC ≤ 400,000 cells/mL, those considered healthy, the average USST was 33.45 °C. For quarters that are considered as infected by subclinical mastitis with SCC > 400,000 cells/mL, the average USST was 35.80 °C. Sathiyabarathi et al. [8] reported a cut-off value of 37.61 °C to consider cows infected by subclinical mastitis. Martins et al. [9] expanded the research to the Santa Inês ewes, observing a slight temperature increase, though non-significant, in the udder surface with subclinical mastitis.

Zaninelli et al. [10] proposed a software approach and reported sensitivity and specificity values of 78.6% and 77.9%, respectively, for mastitis detection at a somatic cell count (SCC) threshold of 200,000 cells/mL. At an SCC threshold of 400,000 cells/mL, sensitivity and specificity were 71.4% and 71.6%, respectively; these values are lower than in other published papers, but they were considered within an acceptable range. More recently, Machado et al. [11] examined the crossbred cows and reported high correlations between udder temperature measurements and somatic cell count (SCC), indicating the potential of thermography as a non-invasive tool for assessing mastitis severity. On the other hand, in the literature, there are references that indicate that IRT is not practical in diagnosing subclinical mastitis as in [12,13]. Velasco-Bolaños et al. [14], who conducted a study on Holstein cows using different USST thresholds varied by milking method, i.e., machine-milked and hand-milked, found that IRT is a clinically valuable method for diagnosing intramammary infections and microbial growth but not subclinical mastitis. The limitations in the diagnostic accuracy of IRT can be attributed to the experimental setup, factors that affect USST such as the humidity of the environment and skin, physiological state and production level of the cow, and time relative to feeding and milking.

The studies cited herein collectively propel the field of mastitis detection forward using infrared thermography, offering valuable insights into its diagnostic capabilities and limitations across various dairy animal populations. However, a predominant reliance on temperature differentials as the primary indicator for subclinical mastitis detection has been observed, and a standardized methodology for detecting subclinical mastitis with IRT still needs to be added to the literature. However, it is increasingly evident that a more nuanced approach is required. Merely focusing on absolute temperature values may not be enough; more attention to critical nuances may be necessary. Thus, there is a growing acknowledgment of the necessity to consider broader aspects, such as thermal patterns and temperature distribution across the mammary gland. Integrating these additional factors into mastitis detection methodologies can significantly enhance their accuracy and effectiveness in precision livestock management endeavors. In this study, we propose an advanced algorithmic approach that integrates thermal imaging with statistical texture analysis and machine learning techniques, aiming to improve the detection accuracy of subclinical mastitis in dairy sheep.

## 2. Materials and Methods

### 2.1. Proposed Methodology

**Figure 1:**
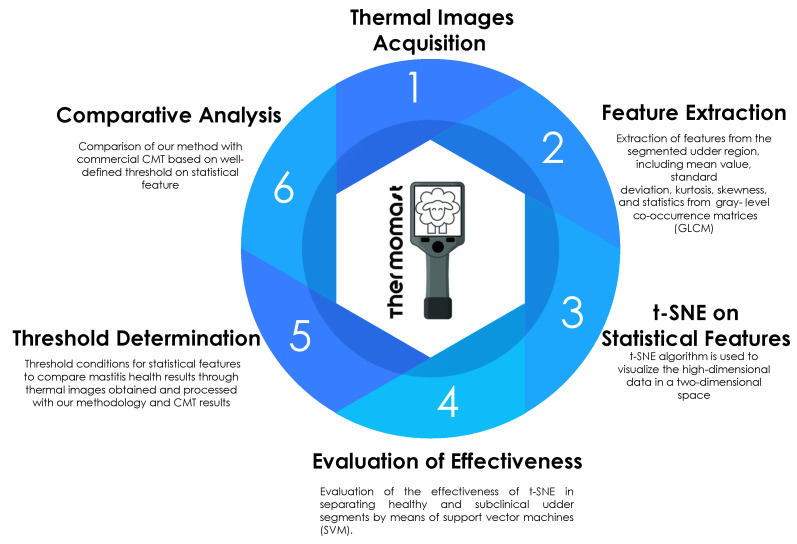
Illustration of the proposed methodology.

We schematically illustrate the steps of our algorithm procedure in Figure 1. The algorithm uses the K-means method to segment thermal images based on color and feature extraction. It allows the user to manually select the region of interest (ROI) for accurate udder segmentation. Through field measurements, we identified signatures of subclinical mastitis and healthy sheep. Discrimination between healthy sheep and sheep with subclinical mastitis was facilitated by the t-SNE technique using machine learning-based classification. We employed different evaluation metrics, such as supervised machine learning algorithms and statistical measures, ensuring the robustness of our approach.

### 2.2. Experimental Setup and Coordination of Measurements for Thermal Images Acquisition

This section outlines the measurement protocol for thermal image acquisition. We chose a UTi260B thermal camera (Uni-Trend Technology Limited, Dongguan, China) [15] with a resolution of 256 × 192 pixels for its cost-effectiveness and high resolution. Notably, the resolution of the thermal cameras used in the literature review is lower [8,11], thereby facilitating the capture of more detailed thermal patterns crucial for our analysis.

Field measurements were carried out in two stages. In November 2022, we initially captured thermal images of udders from 200 sheep distributed across 5 commercial dairy farms in the Tripoli region of Arcadia. In the subsequent phase during Spring 2023, an additional 60 thermal images were incorporated into our dataset. This annotated dataset is publicly accessible (see the Data Availability Statement).

Alongside thermal imaging and on-site clinical assessments by the veterinarian, testing for subclinical mastitis was conducted using the CMT. During field measurements, we encountered several challenges, notably the stress-induced behavior of sheep, which impacted the ease of capturing clear and stable images. To overcome these obstacles and ensure the collected data’s quality, we established rigorous selection criteria focused on image focus, stability, and the absence of motion blur. Adherence to these criteria was paramount in minimizing data variability and enhancing the precision of our subsequent analysis. Detailed explanations of our image selection process and criteria are elaborated upon in Appendix A, ensuring our methodology is replicable and robust.

### 2.3. Segmentation of Udder Thermal Images

**Figure 2:**
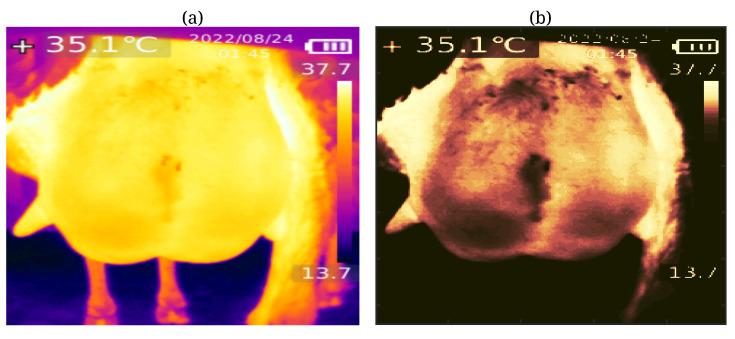
First-stage segmentation results using color-based segmentation with K-means clustering. (**a**) The thermal image shows the color-based segmentation with three clusters (foreground, background, and third cluster). (**b**) The segmented image after K-means clustering, showing the cluster with the highest mean intensity value representing the foreground object of interest.

**Figure 3:**
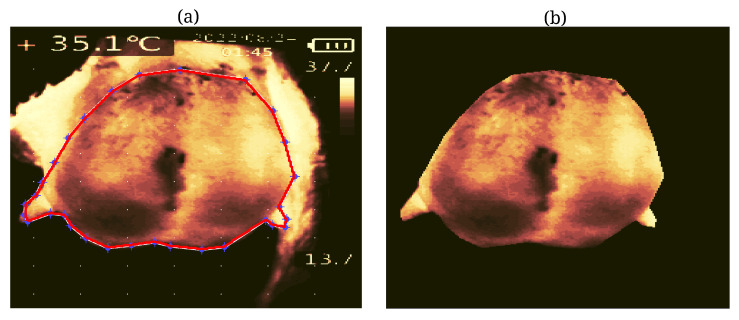
The second stage of segmentation results when the user selects the polygon of interest in the image. (**a**) The red lines indicate the boundaries of the polygon of interest, while the blue stars show the points the user selected to create the polygon. (**b**) The final segmented image after the user-defined polygon selection.

Thermal image segmentation is critical in extracting essential features associated with subclinical mastitis. To address the specific requirements of our project, we developed a two-stage image segmentation methodology tailored to thermal images of sheep udders. In the initial stage, we employed color-based segmentation with K-means clustering [16] to isolate the udder region from the background (see Figure 2). This process involves grouping the pixels of the input image into K clusters based on their color attributes, with K representing a predetermined number of clusters. Subsequently, the clusters are labeled according to the dominant color within each cluster. Assigning each pixel to the cluster with the closest color match enables effective segmentation of the udder region, effectively separating it from the surrounding image. However, it is essential to note that this approach may inadvertently include unwanted regions, such as the leg, in the segmentation output, as illustrated in Figure 2. In instances where automatic segmentation methods prove ineffective or when users wish to focus on specific areas of interest, we implement an interactive method allowing users to delineate a polygonal shape around the desired region of interest (ROI) by clicking on the image to define vertices (see Figure 3). In Figure 3a, the results of the first stage segmentation are depicted, prompting the user to select an ROI by clicking on a series of points (blue stars), automatically forming a polygonal shape (red line). Once the polygon is closed, the function outputs a handle to the ROI object, facilitating the retrieval of vertex coordinates and the creation of a binary mask for the selected area. Subsequently, in Figure 3b, the final segmented image displaying only the udder is presented, obtained by applying the binary mask of the selected region to the original thermal image.

### 2.4. Statistical Texture Analysis and t-SNE Machine Learning Algorithm

Following segmentation, we proceed to extract features from the segmented udder region, encompassing parameters such as mean value, standard deviation, kurtosis, skewness, and statistics derived from gray-level co-occurrence matrices (GLCM) [17]. GLCM, a second-order texture descriptor, quantifies the spatial relationship between pairs of pixels, furnishing insights into the shape, texture, and temperature distribution of the udder. These features are pivotal for early detection of subclinical mastitis, offering a quantitative and objective assessment of thermal patterns imperceptible to the naked eye. Through analysis of these features, we choose to include correlation and contrast for our early-stage subclinical mastitis detection analysis.

Dimensionality reduction is a crucial step in analyzing the features extracted from thermal images for the early detection of subclinical mastitis in dairy animals. This study utilized t-distributed stochastic neighbor embedding (t-SNE), which is a non-linear dimensionality reduction technique well-suited for visualizing complex high-dimensional data. By preserving the local structure of the data, t-SNE can reveal patterns and clusters that may be difficult to discern using other techniques [18]. In particular, we used t-SNE to separate healthy and subclinical samples based on their feature profiles. To evaluate the performance of the proposed method, we incorporated error ellipses to visualize the overlap between the two categories. This allowed us to assess the classification results’ accuracy and reliability and identify potential areas for improvement.

### 2.5. Threshold Determination for Comparative Analysis

**Figure 4:**
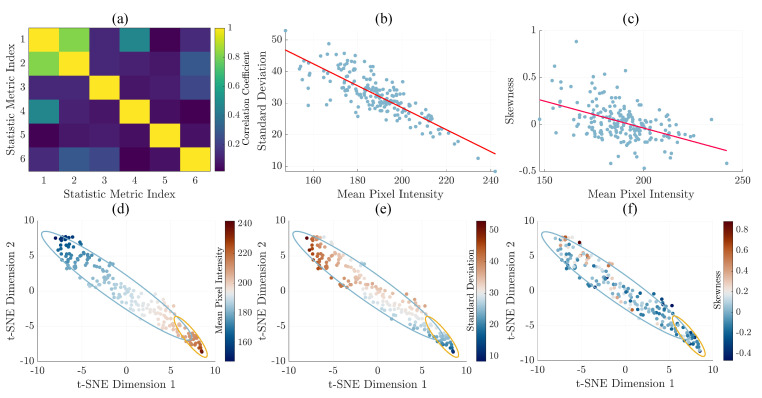
(**a**) Correlation coefficient map displaying interactions among six statistical features: mean pixel intensity (index 1), standard deviation (index 2), kurtosis (index 3), skewness (index 4), correlation (index 5), and contrast (index 6). Correlation scatter plots with linear fit trendline (red line) for mean pixel intensity, standard deviation (**b**), and skewness (**c**). t-SNE plot with error ellipsoids in which mean pixel intensities values (**d**), standard deviation (**e**), and skewness (**f**) are depicted as color-coded markers, corresponding to data from the initial measurement campaign. Note: The ellipses in the t-SNE plots represent confidence intervals of the two clusters.

We performed data analysis on the results of the first measurement campaign to establish threshold conditions for comparative analysis between thermal images and CMT during the second measurement campaign. To achieve this, we calculated the correlation coefficient between six statistical features to understand the interrelationships. As depicted in Figure 4a, mean pixel intensity (index 1) presented a strong correlation with standard deviation (index 2) and a moderate correlation with skewness (index 4).

Subsequently, we plotted correlation scatter plots with linear fit trendlines for mean pixel intensity and standard deviation (Figure 4b) and for skewness (Figure 4c). We observed a decrease in standard deviation and skewness with the increase in mean pixel intensity. This trend could be attributed to regions of higher temperature associated with subclinical mastitis, where pixel intensities are located at the higher end of the temperature scale. Negative skewness suggested that while the majority of temperature measurements were elevated, a tail of lower values extended toward the cooler temperatures found in the rest of the udder. Despite the presence of these cooler regions, the standard deviation was relatively small, indicating that such values were comparatively rare and did not deviate significantly from the mean temperature of the infection site. Furthermore, we utilized a t-SNE plot with feature overlays to visualize mean pixel intensity values (Figure 4d), standard deviation (Figure 4e), and skewness (Figure 4f) as color indicators, representing the statistical feature of each data point. This method facilitated the examination of statistical feature threshold values at the boundaries between healthy and subclinical mastitis cases, denoted by the error ellipsoids.

Through detailed analysis of Figure 4, we determined optimal threshold conditions that effectively differentiate healthy from subclinical samples. These thresholds are 199 for mean pixel intensity, 30 for standard deviation, and −0.05 for skewness. Having established these threshold states, we proceeded to a one-to-one comparison between the results of our method and those from a commercial counterpart; the results are presented in Section 3.2.

## 3. Results and Discussion

### 3.1. Evaluation of t-SNE with Supervised Machine Learning Algorithms

**Figure 5:**
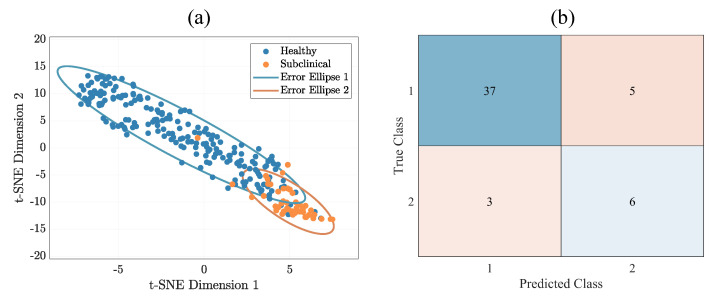
(**a**) The distribution of udders in the two groups (subclinical and healthy) in two-dimensional space was obtained by applying t-SNE to six statistical features. Healthy subjects are shown in blue, while subclinical subjects are shown in orange. The ellipses indicate the 85% confidence interval of the mean and covariance for each group. (**b**) Confusion Matrix illustrating classification results, with TP = 6, TN = 37, FP = 5, and FN = 3.

Figure 5a presents how t-SNE can visualize the six statistical features in a two-dimensional space—the effectiveness of t-SNE in separating healthy and subclinical udders based on statistical features. The blue dots represent healthy udders, while the orange dots represent subclinical udders. The ellipses represent the 85% confidence intervals for each class and allow us to compare the spread of the data visually. The plot separates the two classes, indicating that the statistical features distinguish healthy and subclinical udders [19,20].

To evaluate the effectiveness of the t-SNE algorithm in discriminating healthy and subclinical udders, we use a commonly used machine learning algorithm, the SVM [21]. SVM is a widely used classification algorithm that identifies a hyperplane that separates by maximizing data belonging to different categories. The results of the t-SNE algorithm are used as input features, and the models are trained with an 80/20 ratio between training and testing sets on a dataset with labels for the animals’ health status. Then, the trained models were tested on a separate validation set to evaluate their performance.

The evaluation results showed that SVM can achieve high levels of accuracy in udder classification at 84%. We calculated the confusion matrix (see Figure 5b) in order to calculate sensitivity and specificity, which are widely used performance metrics for assessing the accuracy of a classification model. Sensitivity is related to the probability of correct detection of cases with subclinical mastitis, whereas specificity is related to the probability of correct detection of healthy cases. The following relationships can describe the two metrics [22]:

(1) $$\mathrm{Sensitivity}=\frac{\mathrm{TP}}{\mathrm{FN}+\mathrm{TP}}$$

(2) $$\mathrm{Specificity}=\frac{\mathrm{TN}}{\mathrm{FP}+\mathrm{TN}},$$

wherein TP is when a case of an udder with subclinical mastitis is correctly claimed as a clinical case, TN is when a case of a healthy udder is correctly claimed as a healthy case, FN is when a case of an udder with subclinical mastitis is incorrectly claimed as a healthy case, and FP is when a case of a healthy udder is incorrectly claimed as a clinical case. Sensitivity and specificity, computed from the confusion matrix depicted in Figure 5b, were 67% and 88%, respectively. While the observed sensitivity of our method is moderate, the potential for its enhancement is significant. The 88% specificity in identifying healthy udders establishes a solid baseline for calibration, with a well-defined thermal signature of non-affected tissue providing a key reference point for advancing our algorithm.

### 3.2. Comparative Analysis of Thermal Imaging and California Mastitis Test Results

As we discussed in Section 2.5 at the end of the algorithmic processing of the first phase results, we defined threshold conditions for three statistical features, i.e., 199 for mean pixel intensity, 30 for standard deviation, and −0.05 for skewness. The threshold conditions were used to compare mastitis health results through thermal images obtained and processed with our methodology and CMT results performed in the field by an experienced veterinarian simultaneously with the thermal images. These conditions served as a basis for determining TP, FP, TN, and FN; thus, sensitivity and specificity were determined through Equations (1) and (2). Sensitivity and specificity were used to evaluate and compare the results with those relevant in the literature [7,10].

Our study showcases a superior specificity of 92.5% and sensitivity of 80% compared to previous research on the automatic process of thermal images for subclinical mastitis detection [10]. This work focused on dairy cows, and more directly relevant studies on sheep need to be conducted. Hence, our findings hold promise for the effectiveness of our method in identifying subclinical mastitis in dairy sheep populations. This method offers an alternative metric alongside a confusion matrix (both methods are presented in Table 1) to assess the effectiveness of our method, potentially reducing economic losses within the dairy sheep industry. Our commitment to continuous improvement is demonstrated by our open-access dataset, which invites further expansion and refinement of our diagnostic tool, thereby advancing the detection of subclinical mastitis. The transferability of our tools and methods to related applications holds immense potential for broader impact beyond mastitis detection alone.

## 4. Conclusions

Our comprehensive study has unveiled valuable insights into detecting subclinical mastitis in dairy sheep through a machine learning-enhanced thermal imaging technique. By integrating statistical texture analysis with t-SNE and the SVM algorithm, we achieved a notable classification accuracy of 84%. Our method is further supported by a robust comparison with commercial CMT based on a well-defined threshold on statistical features, showcasing a substantial sensitivity of 80% and a specificity of 92.5%, marking a significant improvement over traditional temperature differential-based methods. This improvement, alongside the ease of implementation and generalizability of our approach, underscores its substantial promise for future commercial application. Our findings, supported by an open-access dataset, encourage further refinement and collaborative efforts to enhance this non-invasive diagnostic tool. Ultimately, this research not only sets the stage for more efficient livestock management strategies but also advances the overarching goals of sustainable farming and animal welfare.

## Figures and Tables

**Table 1:** Evaluation metrics under the two methods presented in this study.

Method	Accuracy	Sensitivity	Specificity
Confusion Matrix for all samples	84%	67%	88%
Threshold-based for samples from second measurement campaign	Not calculated	80%	92.5%

**Figure A1:**
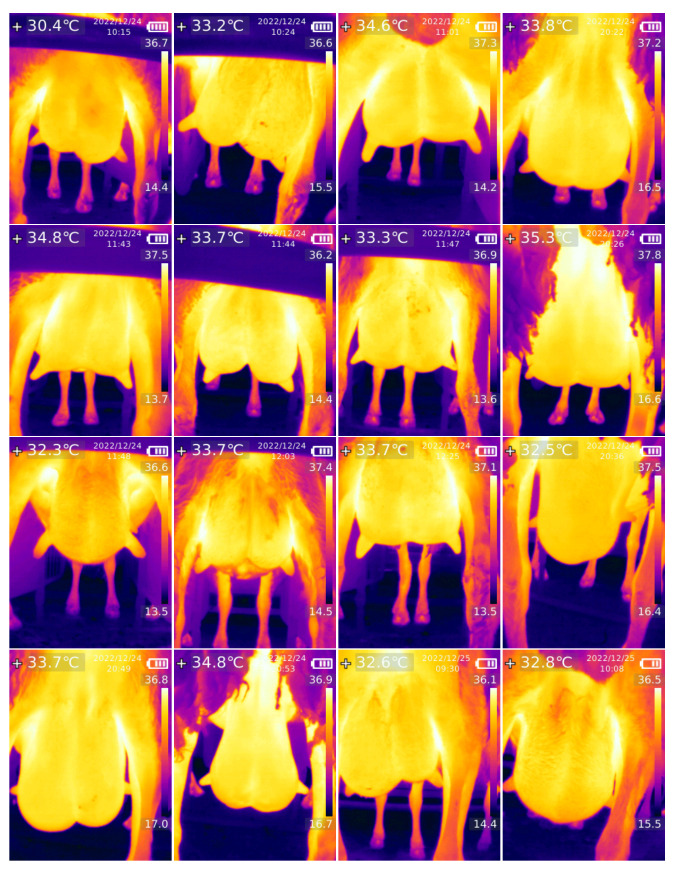
Illustration of images that meet the established selection criteria.

**Figure A2:**
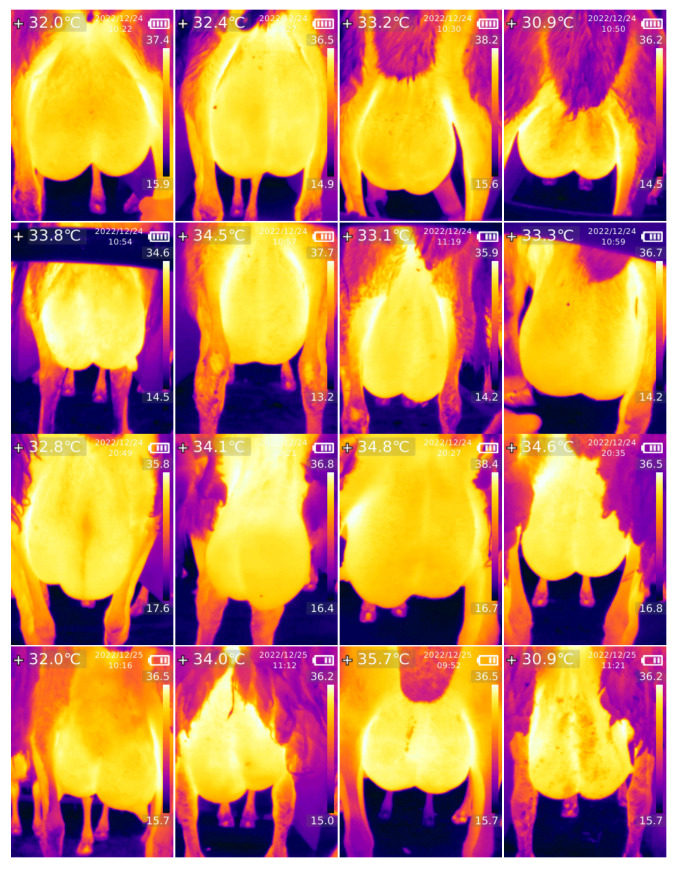
Illustration of images that do not meet the established selection criteria.

## Data Availability

The code developed for this research, due to its commercial value for the company “Power Milk”, is not publicly available. However, interested parties may contact the company directly for inquiries regarding access to the code or collaboration opportunities. Data used in this study are available online for the research community: https://github.com/ChristosTselios/THERMOMAST-Images (accessed on 1 May 2024).

## Abbreviations

The following abbreviations are used in this manuscript:

t-SNE	t-distributed stochastic neighbor embedding
CMT	California mastitis test
IRT	infrared thermography
SVM	support vector machines
USST	udder skin surface temperature

## Appendix A. Image Selection Criteria for Accurate Sheep Udder Analysis

The establishment of selection criteria for the images is crucial due to their potential to mislead conclusions regarding the presence of subclinical mastitis. Images captured from incorrect angles or heights, as well as those with the udder obstructed by stacked legs, can introduce inaccuracies in the analysis and interpretation of the udder’s condition. By implementing specific criteria, such as appropriate angles, clear visibility, and good image quality, the risk of misinterpreting the results is minimized. Ensuring that the selected images adhere to these criteria enhances the reliability and validity of the conclusions drawn from the study, enabling a more accurate assessment of the presence and severity of subclinical mastitis in sheep udders.

The criteria read as follows:

- Angle and Height:
  – Exclude images taken from incorrect angles that do not provide a clear view of the sheep’s udder.
  – Exclude images taken from inappropriate heights that distort the udder’s appearance or visibility.
- Leg Interference:
  – Exclude images where the legs of the sheep are visibly obstructing or overlapping with the udder region.
  – Avoid images where the udder contour is affected by the presence of legs.
- Image Quality:
  – Select images with good overall image quality, clarity, and resolution.
  – Consider excluding images with excessive noise, blurriness, or artifacts that may affect the accuracy of analysis.
- Udder Visibility:
  – Prioritize images where the udder is prominently visible and occupies a significant portion of the image frame.
  – Exclude images where the udder is partially or completely obscured by other body parts or objects.
- Consistency:
  – Maintaining consistency in image selection criteria ensures the dataset is uniform, allowing for reliable analysis and comparisons. Inconsistent image quality can lead to varied results and misinterpretations [23].

Figure A1 demonstrates proper angles and heights, clear visibility of the udder, minimal leg interference, high image quality, and clear visibility. The sheep’s legs are open in these images, providing a clear view of the udder. Even when the legs are not as open, they do not interfere with the measurement, ensuring accurate temperature readings.

The images that fail to meet the selection criteria due to incorrect angles, inappropriate heights, leg interference, poor image quality, or obstructed udder visibility are presented in Figure A2. For instance, the leg is in front of the udder or touches the udder, resulting in increased temperature readings that can mislead the analysis. It is challenging to control these factors during measurement because we aim to give the animals as much freedom as possible to avoid stressing them. By opening their legs unnaturally, the stress on the animals can be increased, leading to false measurements due to stress-induced temperature changes.
